# Significant detection of new germline pathogenic variants in Australian Pancreatic Cancer Screening Program participants

**DOI:** 10.1186/s13053-021-00190-1

**Published:** 2021-08-16

**Authors:** Krithika Murali, Tanya M. Dwarte, Mehrdad Nikfarjam, Katherine M. Tucker, Rhys B. Vaughan, Marios Efthymiou, Allison Collins, Allan D. Spigelman, Lucinda Salmon, Amber L. Johns, David B. Williams, Martin B. Delatycki, Thomas John, Alina Stoita

**Affiliations:** 1 Department of Clinical Genetics, Austin Health, Heidelberg, VIC 3084 Australia; 2 Australian Pancreatic Cancer Genome Initiative, Garvan Institute of Medical Research, Darlinghurst, NSW 2010 Australia; 3 Hereditary Cancer Centre, Prince of Wales Hospital, Randwick, NSW 2031 Australia; 4 Department of Gastroenterology, St Vincent’s Hospital, Darlinghurst, NSW 2010 Australia; 5 Division of Surgery, Austin Health, Heidelberg, VIC 3084 Australia; 6 University of New South Wales, Prince of Wales Clinical School, Randwick, NSW 2031 Australia; 7 Department of Gastroenterology, Austin Health, Heidelberg, VIC 3084 Australia; 8 Clinical Trials Unit, Olivia Newton John Cancer and Wellness Centre, Austin Health, Heidelberg, VIC 3084 Australia; 9 Cancer Genetics Unit, The Kinghorn Cancer Centre, St Vincent’s Hospital, Darlinghurst, NSW 2010 Australia; 10 Peter MacCallum Cancer Centre, Parkville, VIC 3000 Australia

**Keywords:** Pancreatic cancer, Cancer screening, Genetics, Genetic testing, Pathogenic variant, Hereditary Cancer syndromes

## Abstract

**Background:**

The Australian Pancreatic Cancer Screening Program (APCSP) offers endoscopic ultrasound surveillance for individuals at increased risk of pancreatic ductal adenocarcinoma (PDAC) with all participants requiring assessment by a Familial Cancer Service before or after study enrolment.

**Methods:**

Individuals aged 40–80 years (or 10 years younger than the earliest PDAC diagnosis) were eligible for APCSP study entry if they had 1) ≥ two blood relatives with PDAC (at least one of first-degree association); 2) a clinical or genetic diagnosis of Hereditary Pancreatitis or Peutz-Jeghers syndrome irrespective of PDAC family history; or 3) a known PDAC predisposition germline pathogenic variant (*BRCA2, PALB2, CDKN2A,* or Lynch syndrome) with ≥one PDAC-affected first- or second-degree relative.

Retrospective medical record review was conducted for APCSP participants enrolled at the participating Australian hospitals from January 2011 to December 2019. We audited the genetic investigations offered by multiple Familial Cancer Services who assessed APCSP participants according to national guidelines, local clinical protocol and/or the availability of external research-funded testing, and the subsequent findings. Descriptive statistical analysis was performed using Microsoft Excel.

**Results:**

Of 189 kindreds (285 participants), 50 kindreds (71 participants) had a known germline pathogenic variant at enrolment (*BRCA2 n* = 35, *PALB2 n* = 6, *CDKN2A* n = 3, *STK11* n = 3, *PRSS1 n* = 2, *MLH1 n* = 1). Forty-eight of 136 (35%) kindreds with no known germline pathogenic variant were offered mutation analysis; 89% was clinic-funded, with increasing self-funded testing since 2016. The relatively low rates of genetic testing performed reflects initial strict criteria for clinic-funded genetic testing. New germline pathogenic variants were detected in five kindreds (10.4%) after study enrolment (*BRCA2* n = 3 kindreds, *PALB2* n = 1, *CDKN2A* n = 1). Of note, only eight kindreds were reassessed by a Familial Cancer Service since enrolment, with a further 21 kindreds identified as being suitable for reassessment.

**Conclusion:**

Germline pathogenic variants associated with PDAC were seen in 29.1% of our high-risk cohort (55/189 kindreds; 82/285 participants). Importantly, 10.4% of kindreds offered genetic testing were newly identified as having germline pathogenic variants, with majority being *BRCA2*. As genetic testing standards evolve rapidly in PDAC, 5-yearly reassessment of high-risk individuals by Familial Cancer Services is warranted.

## Background

Around 3600 new cases of pancreatic cancer (PC) were diagnosed in Australia in 2019 and the incidence is rising [1]. Approximately 95% of PC are pancreatic ductal adenocarcinomas (PDAC) [2]. PDAC has a poor prognosis as most patients present with advanced or metastatic disease [2]. Five-year survival rates are greatly influenced by the disease stage at the time of diagnosis [3].

Approximately 10% of PDAC clusters in families [4]. Eighty to 90% of familial PDAC cases do not yet have their genetic susceptibility identified [5]. However a family history of PDAC carries a 2.3 to 32-fold increased risk, depending upon the number of family members affected [5]. Familial pancreatic cancer (FPC) is defined as a family that contains at least two first-degree relatives affected by PDAC where the family history does not suggest a known cancer-predisposition syndrome, or where no causative germline pathogenic variant is identified [6]. Approximately 5–10% of patients with PDAC meet the criteria for FPC [7].

Only 10–20% of high-risk kindreds have a known heritable gene mutation [8–10] with the majority due to germline pathogenic variants in *BRCA1* [11, 12], *BRCA2* [12–14], *CDKN2A* [15], the mismatch repair genes (*MLH1*, *MSH2*, *MSH6, PMS2),* [8, 16, 17]*,* Hereditary Pancreatitis genes [18] *(SPINK1, PRSS1)*, *STK11* [19], and *TP53* [17, 20]. More recently, *PALB2* [21] and *ATM* [10] germline pathogenic variants have been identified in 3–5% of familial PDAC cases.

*BRCA2* germline pathogenic variants are found in 3.5 to 17% of families with PDAC [8, 12, 13] with the *BRCA2 c.5946del* Jewish Founder Mutation enriched in cohorts with significant Ashkenazi Jewish ancestry [22–24]. However, the penetrance of PDAC in those with *BRCA2* germline pathogenic variants is low, with 95% of carriers not developing PDAC during their lifetime [25]. *ATM* germline pathogenic variants are the next most prevalent, present in 2–3% of PDAC families [26] but the PDAC penetrance is less clear. Other relevant germline pathogenic variants are less common and found in ≤1% of affected kindreds [27]. Although rare, Peutz-Jeghers syndrome (caused by germline pathogenic variants in *STK11*) has the highest associated cumulative lifetime PDAC risk of 36% [19]. Those with *CDKN2A* germline pathogenic variants have up to a 17% lifetime risk [28].

Recent studies of germline pathogenic variants in individuals with PDAC unselected for family history, age or other high-risk features have demonstrated a 3.5 to 13% germline mutation detection rate in established PDAC-associated genes [10, 27, 29–32]. This is particularly relevant as traditional family and personal history criteria can miss germline pathogenic variants in PDAC patients [10, 27, 33]. Based on this accumulating evidence, American Society of Clinical Oncology (ASCO) guidelines recommend consideration of germline genetic testing in all individuals diagnosed with PDAC [34]. Identifying a germline pathogenic variant in PDAC is important as it may allow targeted therapies such as Poly ADP-ribose polymerase (PARP) inhibitors in those with *BRCA1/BRCA2* pathogenic variants [35, 36] and immunotherapy in Lynch syndrome [37]. Additional benefits include consideration of second primary cancer risk in survivors and identification of ‘at-risk’ relatives who may then seek appropriate surveillance.

PC screening with endoscopic ultrasound (EUS) and magnetic resonance imaging (MRI) in high-risk individuals can potentially reduce mortality by detecting PDAC at an earlier stage or its precursor lesions [38–42]. The average lifetime risk of developing PDAC is too low to advocate population screening [41], therefore the identification of high-risk individuals is key to ensuring PC screening is targeted to those who may derive the greatest benefit. Genetic information can help identify suitable high-risk individuals and can help personalise PC surveillance (e.g. individuals with Peutz-Jeghers syndrome commencing surveillance at a younger age) [41]. As a result of the growing importance of genetics, the American College of Gastroenterology recommends that genetic counselling be standard of care for individuals entering PC screening programs [40].

Given the continued controversy about the clinical utility and cost-effectiveness of PC screening, screening should only be performed in a research setting in specialised clinical centres, in line with Cancer of the Pancreas Surveillance (CAPS) consortium recommendations [41]. The Australian Pancreatic Cancer Screening Program (APCSP) is a research program that offers EUS and MRI surveillance for individuals at increased risk of developing PDAC. All high-risk individuals require mandatory assessment by a Familial Cancer Service as part of study participation. This provides us with a unique opportunity to describe the familial characteristics as well as the genetic testing pathways and outcomes of a high-risk cohort in the Australian context.

## Method

The aim of this study was to summarise the familial characteristics and genetic testing outcomes of high-risk individuals and kindreds participating in the APCSP.

### Recruitment

The APCSP commenced at St Vincent’s Hospital, Sydney in 2011 and at Austin Health, Melbourne in 2015. The study received institutional ethics approval at both sites (Approval numbers 10/055 and H2013/04954). Recruitment of high-risk individuals was possible via multiple pathways and was described in detail previously [43]. All participants provided written informed consent.

All participants were assessed to determine eligibility by the clinical research coordinator before enrolment. Individuals aged 40–80 years (or 10 years younger than the earliest PDAC diagnosis in the family), who met one of the following criteria were eligible for study entry: 1) two or more blood relatives with PDAC (including at least one of first-degree association); 2) a clinical or genetic diagnosis of Hereditary Pancreatitis or Peutz-Jeghers syndrome irrespective of PDAC family history; or 3) carrier of a known PDAC predisposition germline pathogenic variant (*BRCA2, PALB2, CDKN2A,* or Lynch syndrome *(MLH1, PMS2, MSH6, MSH2 or EPCAM* deletion)) with at least one PDAC-affected first- or second-degree relative.

### Genetic counselling and testing

Prerequisite formal genetic counselling was completed either before or after screening program enrolment. Individuals meeting FPC criteria who had not completed genetic counselling prior to enrolment were referred to a Familial Cancer Service for assessment before their EUS. If clinically indicated, genetic testing was offered to either the screening trial participant or their close affected relative. National Australian guidelines and clinical protocols to determine eligibility for clinic-funded testing were applied, which varied over time, but initially required a greater than 10% likelihood of identifying a germline pathogenic variant or the presence of a germline pathogenic variant in a biological relative. Those not eligible for clinic-funded testing were able to consider self-funded genetic testing. Some individuals underwent external research-funded genetic testing, independent of the screening program, if the Familial Cancer Service identified that they met inclusion criteria.

The decision to facilitate genetic testing was made on an individual basis and included any genetic investigation for an inherited cancer predisposition syndrome, such as immunohistochemistry of the mismatch repair proteins (MMR IHC) or mutation analysis (e.g. Jewish Founder Mutation screen; predictive testing; single gene, gene panel or whole exome sequencing (WES)). Research results were confirmed in a National Association of Testing Authorities (NATA), Australia authorised laboratory. All clinical genetic testing was performed in accredited Australian or international clinical laboratories. All reported genetic test results were confirmed by acquisition of clinical test reports from the appropriate Familial Cancer Service.

### Data analysis

Data from participants enrolled at St Vincent’s Hospital, Sydney from January 2011 to December 2019 and at Austin Health, Melbourne from January 2015 to December 2019 were included in the current analysis. All participants were required to complete a detailed baseline personal and family health history questionnaire. Participant reported family history was cross-checked with clinical correspondence and de-identified pedigrees provided by the Familial Cancer Service. Confirmation of PDAC diagnosis was obtained through verification of pathology reports, death certificates or cancer registry databases with reported family history corroborated by multiple relatives in unconfirmed cases. Genetic testing is reported for either the participant (if tested) or their affected relative (if uninformative), with the findings updated as new germline pathogenic variants were identified. Descriptive statistical analysis was performed using Microsoft Excel. Key case vignettes have been included to demonstrate genetic testing approaches and family outcomes of newly-identified germline pathogenic variants.

## Results

### Participant demographics

Two-hundred and eighty-five participants, comprising 189 kindreds, enrolled in the APCSP had completed genetic counselling through a Familial Cancer Service at the time of analysis. Participant demographics are summarised in Table 1. Participant family history demographics were updated to account for any new PC diagnoses detected through screening at the time of analysis (*n* = 11 participants, three kindreds).

**Table 1:** Participant demographics

Criteria		Number (%)
**Gender**	Male	107 (37.5)
	Female	178 (62.5)
**Ethnicity**	White/Caucasian (non-Jewish)	249 (87.4)
	White/Caucasian (Jewish)	19 (6.6)
	Middle Eastern (non-Jewish)	4 (1.4)
	Middle Eastern (Jewish)	1 (0.4)
	Asian	2 (0.7)
	Hispanic	2 (0.7)
	Other/Unknown	8 (2.8)
		Mean (Range)
**Age**	Overall	55.5 (27-79y)
	Male	56.4 (30-78y)
	Female	54.9 (27-79y)
**PC Family History**	**Number of participants (Kindreds)**	**Youngest PC diagnosis in kindred (Years)** **Mean (range)**
FPC	203 (134)	58 (21–84)
*BRCA2* GPV + ≥1FDR/SDR PC	55^b^ (38)	59 (39–84)
*PALB2* GPV + ≥1FDR/SDR PC	12^c^ (7)	50 (25–76)
*CDKN2A* GPV + ≥1FDR/SDR PC	9 (4)	55 (43–71)
Lynch syndrome+ ≥ 1FDR/SDR PC	1 (1)	80
Hereditary Pancreatitis	2 (2)	54
Peutz-Jeghers syndrome^a^	3 (3)	N/A

^a^clinical diagnosis or *STK11* GPV; ^b^ n = 2 at 50% risk; ^c^ n = 1 at 50% risk; *GPV* germline pathogenic variant; *FDR* first-degree relative; *SDR* second-degree relative

### Genetic investigations and prerequisite genetic counselling

**Fig. 1:**
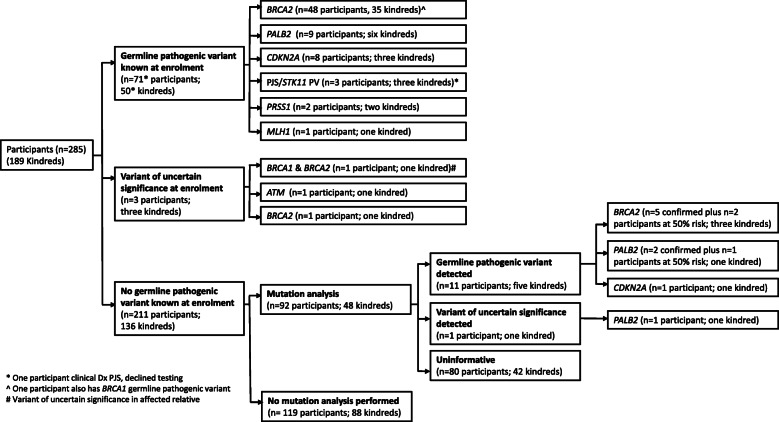
Consort diagram of study participant genetic testing pathways

The genetic testing pathway for all study participants is depicted in a consort diagram (Fig. 1). At the time of screening program enrolment, 70/285 (24.6%) participants from 50/189 (26.5% kindreds) were identified as a germline pathogenic variant carrier for a known PDAC susceptibility gene and one participant had a clinical diagnosis of Peutz-Jeghers syndrome but declined genetic testing. Additionally, three kindreds (*n* = 2 participants; *n* = 1 affected first-degree relative) had a variant of uncertain significance in a known PDAC susceptibility gene (Fig. 1) had a clinical diagnosis of Peutz-Jeghers syndrome but declined genetic testing. Additionally, three kindreds (*n* = 2 participants; *n* = 1 affected first-degree relative) had a variant of uncertain significance in a known PDAC susceptibility gene (Fig. 1).

**Fig. 2:**
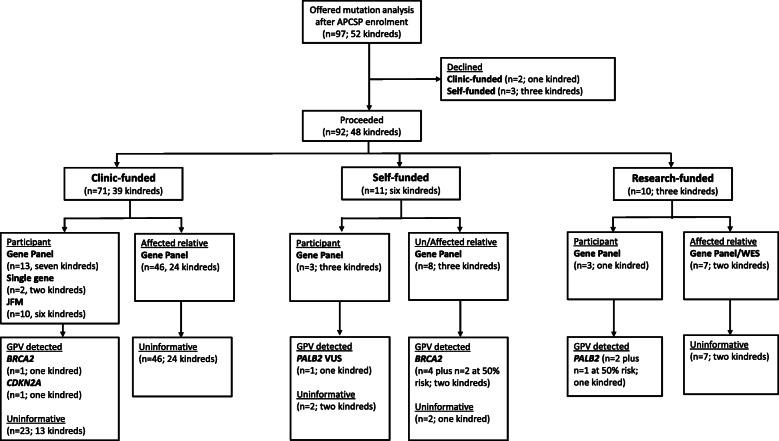
Outcome of mutation analysis in kindreds without a known germline pathogenic variant at enrolment

Of the 136 kindreds with no known germline pathogenic variant at enrolment, 52 were offered genetic testing (either clinic, external research or self-funded) during their genetics consultation. At least one individual from 48 kindreds proceeded, however, three unaffected participants declined self-funded testing and one kindred declined clinic-funded testing on an affected first-degree relative’s stored DNA. The type of investigation, individual tested and funding source for the 48 kindreds that proceeded with mutation analysis are shown in Fig. 2. In six of these kindreds, updated testing was performed due to a new diagnosis; in response to a relative’s genetic test result or the family was reviewed when a younger relative enrolled in the study.

**Fig. 3:**
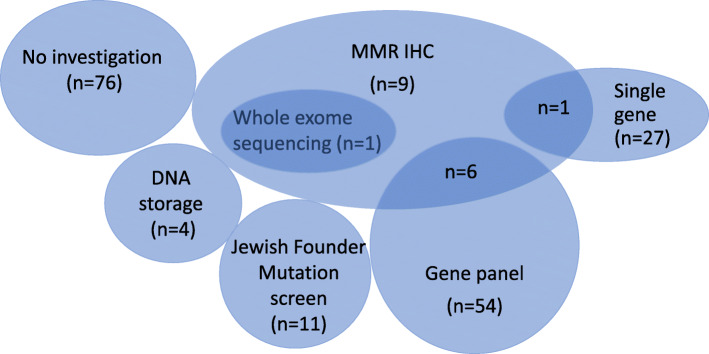
Venn diagram of genetic testing offered to APCSP kindreds (*n* = 189)

Clinic-funded MMR IHC to evaluate the likelihood of Lynch syndrome was arranged for 17 kindreds. All specimens showed preserved staining. This was the only investigation offered for nine kindreds, with the remaining eight kindreds undergoing additional mutation analysis (Fig. 3). DNA from a close affected relative for an additional four kindreds was stored without initiation of mutation analysis. An affected relative for one kindred was offered testing but died prior to blood collection. Genetic investigations performed in three participants unrelated to the PDAC family history (e.g. negative predictive test from maternal side when paternal family history meets FPC criteria) were excluded from analysis. Mutation analysis was performed for at least one individual in 100/189 (52.9%) kindreds. In some cases, mutation analysis was performed for an affected or unaffected relative (30 and 1 kindreds, respectively) rather than the screening participant themselves. However, one or more screening participant(s) from the remaining 69 kindreds directly underwent mutation analysis.

**Fig. 4:**
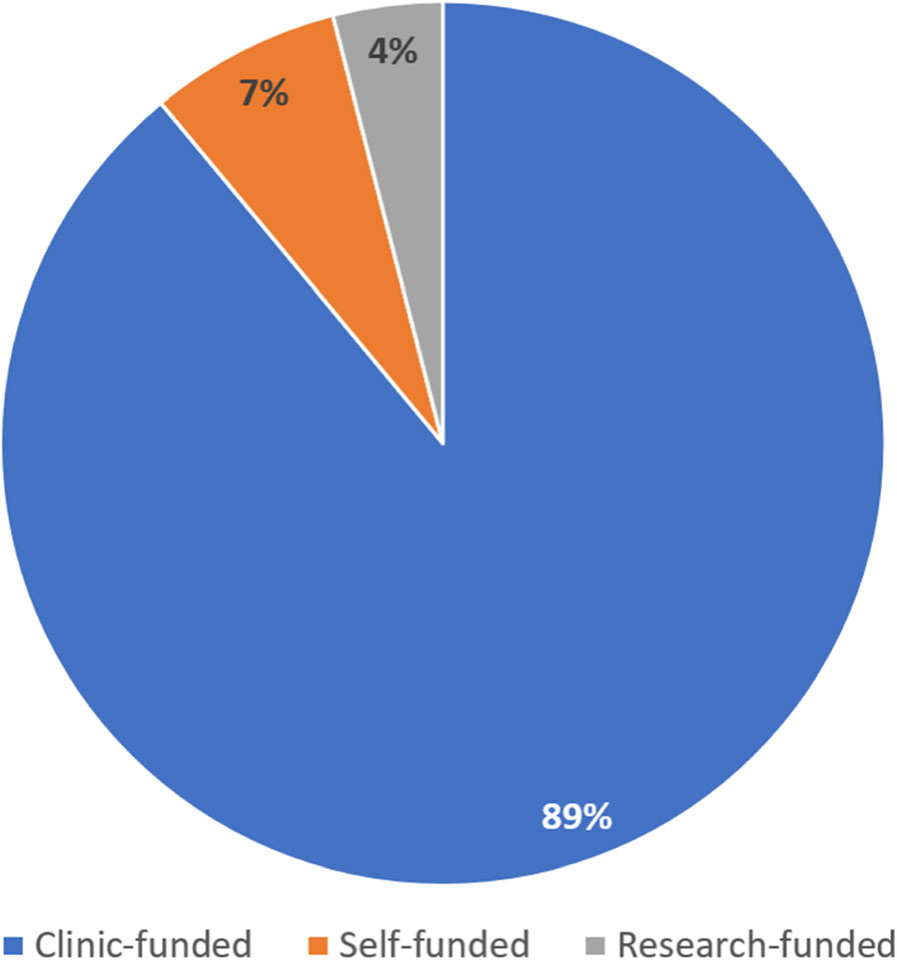
Funding source for primary genetics investigation(s) in tested kindred (*n* = 100)

The funding source for mutation analysis in the 100 tested kindreds (*n* = 166 participants) is shown in Fig. 4. Eighty-nine kindreds (*n* = 151 participants) had clinic-funded genetic testing, seven kindreds (*n* = 7 participants) had self-funded testing and four kindreds (*n* = 8 participants) underwent external research-funded testing. Unsurprisingly, self-funded testing was only observed since 2016 (seven kindreds).

**Fig. 5:**
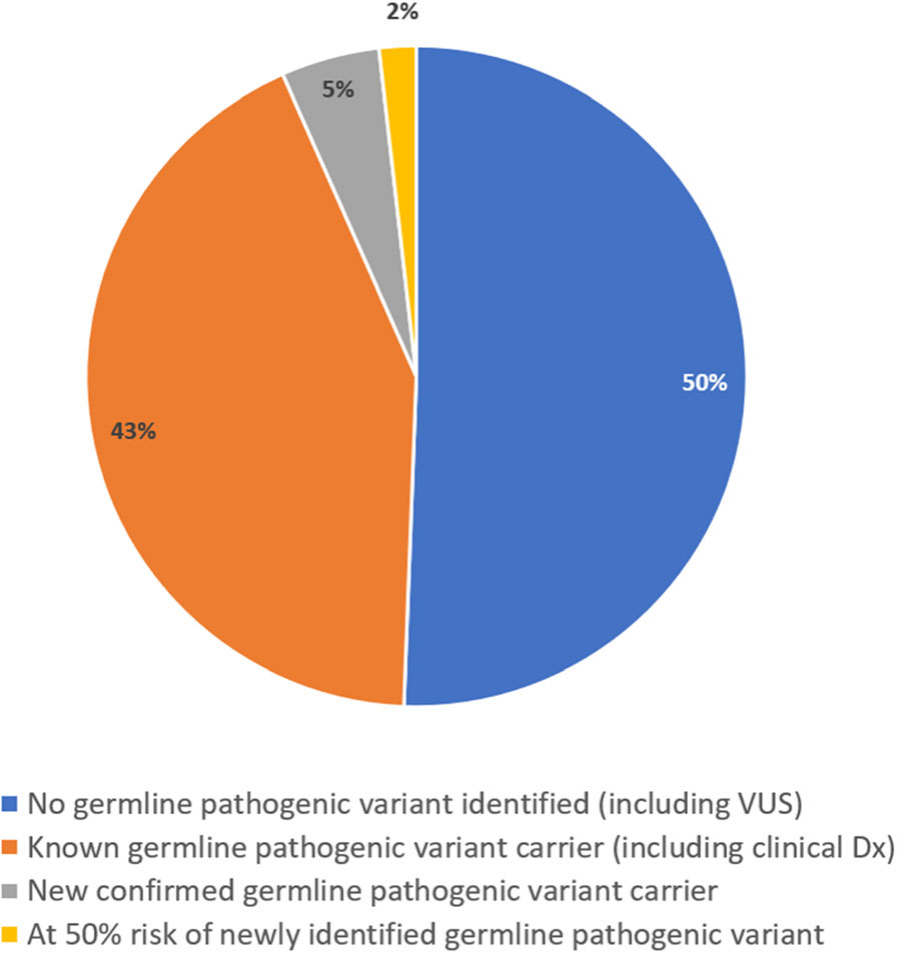
Germline pathogenic variant status of all tested participants (n = 166)

**Fig. 6:**
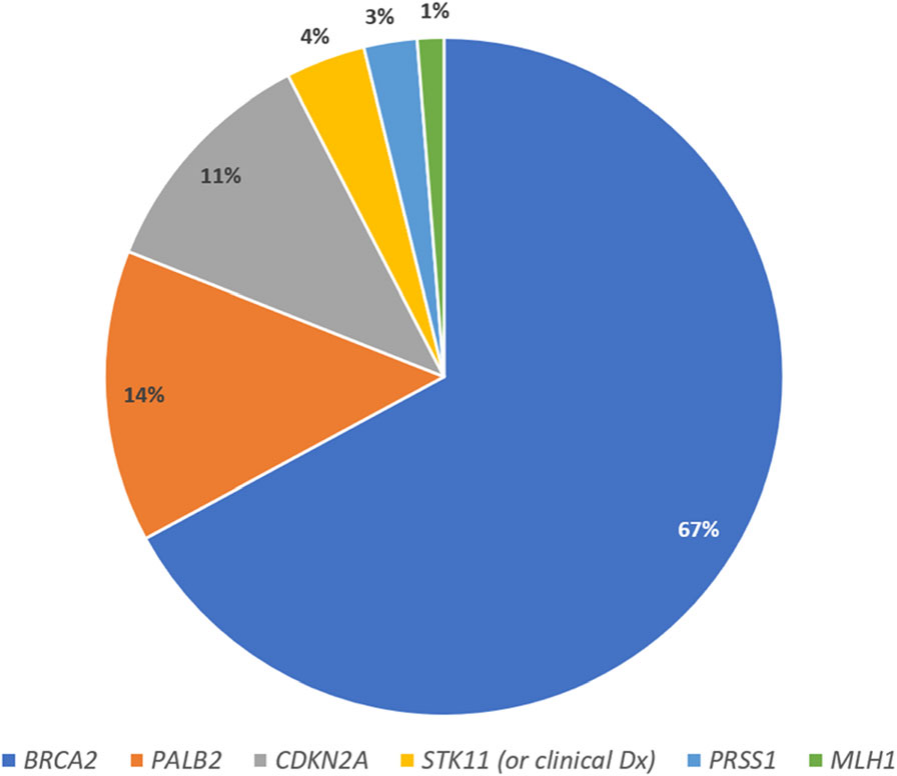
Distribution of germline pathogenic variants in known and newly-found carriers (*n* = 79)

Interestingly, new germline pathogenic variants (Class 4 or 5) were detected in 5/48 (10.4%) kindreds (*n* = 3 *BRCA2*; n = 1 *PALB2*; n = 1 *CDKN2A*). In addition, one participant had a newly identified *PALB2* variant of uncertain significance. Three participants (from three separate kindreds) at 50% risk of a newly-identified germline pathogenic variant had not undergone predictive testing at the time of analysis. The complete cohort of participants and kindreds with a germline pathogenic variant is shown in Table 2. The germline pathogenic variant status of all tested participants is summarised in Figs. 5 and 6.

**Table 2:** All germline pathogenic variants (PV) and variants of uncertain significance (VUS) identified in screening participants

Gene	HGVS Nomenclature	Molecular consequence	Number of participants	PHx Cancer	FHx PDAC
*ATM*	c.1444A > C	VUS	n = 1 Female	Dx 47 breast	1FDR;1SDR
*BRCA1*	c.14C > T ^a^	VUS	n = 1 Female^a^	–	2FDR; 1SDR
	c.2475delC	Pathogenic; Frameshift/Truncating mutation	n = 1 Female^b^	Dx 42/54 breast; Dx 48 fallopian tube	1FDR
*BRCA2*	c.250C > T	Pathogenic; Nonsense	n = 1 Female	Dx 49 breast	1FDR
	c.750_753del4	Pathogenic; Deletion	n = 1 Female	–	2SDR
	c.971G > C^a^	VUS	n = 1 Female^a^	–	2FDR; 1SDR
	c.2808_2811del	Pathogenic; Frameshift/Truncating mutation	n = 1 Male (at 50% risk)	–	2FDR (siblings)
	c.3847_3848delGT	Pathogenic; Frameshift/Truncating mutation	n = 1 Male	–	2FDR
	c.4405_4409del	Pathogenic; Frameshift/Truncating mutation	n = 1 Female	Dx 53 follicular thyroid; Dx 63 breast	1FDR; 1TDR
	c.4478_4481del	Pathogenic; Frameshift/Truncating mutation	n = 1 Female	Dx 37/49 breast	1FDR
	c.4544del	Pathogenic; Frameshift/Truncating mutation	n = 1 Male	Dx 42/43 melanoma	1FDR
	c.4587dupG	Pathogenic; Frameshift/Truncating mutation	n = 1 Male	–	5FDR
	c.5238dup	Pathogenic; Nonsense	**Kindred 1**		
			n = 1 Female	Dx 57/58 breast	1FDR; 1SDR
			**Kindred 2**		
			*n* = 1 Female^c^	Dx 55 PDAC on study; Dx56 CRC	1FDR
			n = 1 Female	-	2FDR
			n = 1 Male	-	2FDR
	c.5303-5304delTT	Pathogenic; Frameshift/Truncating mutation	n = 1 Female	–	1FDR
	c.5681dupA	Pathogenic; Truncating mutation	**Kindred 1**		
			n = 1 Female^c^	Dx 48 PDAC on study; Dx49 gastric	1FDR; 1SDR
			n = 2 Males	-	2FDR^c^; 1SDR
			n = 1 Female	-	1FDR; 2SDR^c^
			n = 1 Female (at 50% risk)	-	2FDR^c^; 1SDR
	c.5682C > G	Pathogenic; Truncating mutation	n = 1 Male	Dx 58 liver	1FDR
	c.5946del (Jewish founder)	Pathogenic; Frameshift/Truncating mutation	**Kindred 1**		
			n = 1 male	Dx 64 prostate	1FDR
			n = 1 female	-	1FDR
			n = 1 female	-	1SDR
			**Kindred 2**		
			n = 1 female	Dx 45 DCIS	1SDR
			**Kindred 3**		
			n = 1 Female^b^	Dx 42/54 breast; Dx 48 fallopian tube	1FDR
			**Kindred 4**		
			n = 1 Female	-	1SDR; 3TDR
			n = 1 Female	Dx 65 ovarian	1SDR; 3TDR
			**Kindred 5**		
			n = 1 Male	-	1FDR
			**Kindred 6**		
			n = 1 female	-	1TDR
			n = 2 males	-	1SDR
			**Kindred 7**		
			n = 1 male	Dx 55 brain; Dx 60 prostate	1FDR; 1SDR
			**Kindred 8**		
			n = 1 Male	-	1FDR
			n = 1 Female	Dx 34 CRC	1FDR
	c.6405_6409delCTTAA	Pathogenic; Frameshift/Truncating mutation	n = 1 Male	–	1FDR
	c.7505G > A	VUS	n = 1 Female	Dx 32 breast	1FDR; 1SDR
	c.7757G > A	Pathogenic; Nonsense	n = 1 Male	–	1FDR
	c. 7806-2A > G	Pathogenic; Splice acceptor	n = 1 Male	–	1FDR
	c.7976 + 5G > C	Likely pathogenic; Splicing disruptor	n = 1 Female	–	1SDR
	c.7977-1G > C	Pathogenic; Splice acceptor	n = 1 Male	–	1SDR
	c.7988A > T	Pathogenic; Missense	n = 1 Female	Dx 49 breast	1FDR
	c.8167G > C	Pathogenic; Missense	n = 1 Female	Dx 39 breast	1FDR
	c.8575del	Pathogenic; Frameshift/Truncating mutation	**Kindred 1**		
			n = 1 Male	Dx 75 RCC	1FDR
			n = 1 Female	Dx 62 breast	1FDR
			n = 1 Male	-	1SDR
			**Kindred 2**		
			n = 1 Female	-	1FDR
	c.9154C > T	Pathogenic; Missense	n = 1 Female	–	1FDR; 1SDR
	c.9294C > G	Pathogenic; Truncating mutation	**Kindred 1**		
			n = 1 Male	*-*	1FDR
			n = 1 Female	Dx 46 breast	1FDR
	c.9371A > T	Pathogenic; Missense	n = 1 Female	Dx 44 breast	1SDR
	c.9924C > G	Pathogenic; Truncating mutation	**Kindred 1**		
			n = 1 Female	Dx 43 breast	2TDR
			n = 1 Female	Dx 33/40 breast	1SDR; 1TDR
	Exon 14–16 deletion	Pathogenic; Deletion	n = 1 Male	–	1FDR
CDKN2A	c.47 T > G p.(Leu16Arg)	Pathogenic; Missense	n = 1 Female	Dx 45 melanoma	1FDR; 1SDR
	c.95 T > C	Pathogenic; Missense	**Kindred 1**		
			n = 1 Female	-	3FDR
			**Kindred 2**		
			N = 1 Male n = 1	Dx 30 melanoma	2SDR
			Female	Dx 21 melanoma	2SDR
			n = 1 Female	-	1FDR; 1SDR
MLH1	c.1758delC	Pathogenic; Truncating mutation	n = 1 Male	Dx 37 BCC; Dx 40 CRC	1FDR
PALB2	c.487_488del	Pathogenic; Frameshift/Truncating mutation	**Kindred 1**		
			n = 1 Female	Dx 49 breast	1FDR; 2SDR
			n = 1 Female (at 50% risk)	-	1FDR; 2SDR
			n = 1 Male	-	1FDR; 2SDR
	c.2257C > T	Pathogenic; Nonsense	**Kindred 1**		
			n = 1 Female	-	1FDR; 1SDR
			**Kindred 2**		
			n = 1 Female	-	1FDR
	c.3113G > A	Pathogenic; Nonsense	n = 1 Male	Dx 40 melanoma	1FDR
			n = 1 Female	Dx 52 BCC	
	c.3209 T > C	VUS	n = 1 Female	Dx 55 breast	2FDR
	c.3362delG	Pathogenic; Frameshift	**Kindred 1**		
			n = 1 Male	-	1SDR
			n = 1 Female	Dx 28 melanoma; Dx 42 breast; Dx 55 SCC/BCC	1FDR
	c.3426_3429del	Pathogenic; Frameshift	n = 1 Female	–	1FDR; 1SDR
PRSS1	c.86A > C	Pathogenic; Missense	n = 1 Male	Dx chronic pancreatitis	Nil PDAC; 1FDR chronic pancreatitis
	c.86A > T	Pathogenic; Missense	n = 1 Female	Dx chronic pancreatitis	1FDR; 2SDR
STK11	c.179dup	Pathogenic, Nonsense	n = 1 Male	–	De novo PV
	Exon 2–9 deletion	Pathogenic; Deletion	n = 1 Female	–	De novo PV

*FDR* first-degree relative; *SDR* second-degree relative; *TDR* third-degree relative; *CRC* colorectal cancer; *DCIS* ductal carcinoma in situ; *BCC* basal cell carcinoma; *SCC* squamous cell carcinoma; *RCC* renal cell carcinoma. ^a^ same participant; both VUS identified in affected FDR; ^b^ same participant; ^c^ diagnosed PDAC on study

Overall, 119 participants (88 kindreds) did not undergo any mutation analysis despite meeting FPC criteria, typically as no affected relative was available to test. These participants were seen when Australian national consensus guidelines excluded unaffected individuals from clinic-funded genetic testing. At the time of analysis, 23 individuals from eight kindreds have undergone Familial Cancer Service reassessment (Table 3). A further 21/134 (16%) untested FPC kindreds may be suitable for Familial Cancer Service reassessment due to recent changes in clinical practice and the relevant services have been notified by the authors Table 4).

**Table 3:**
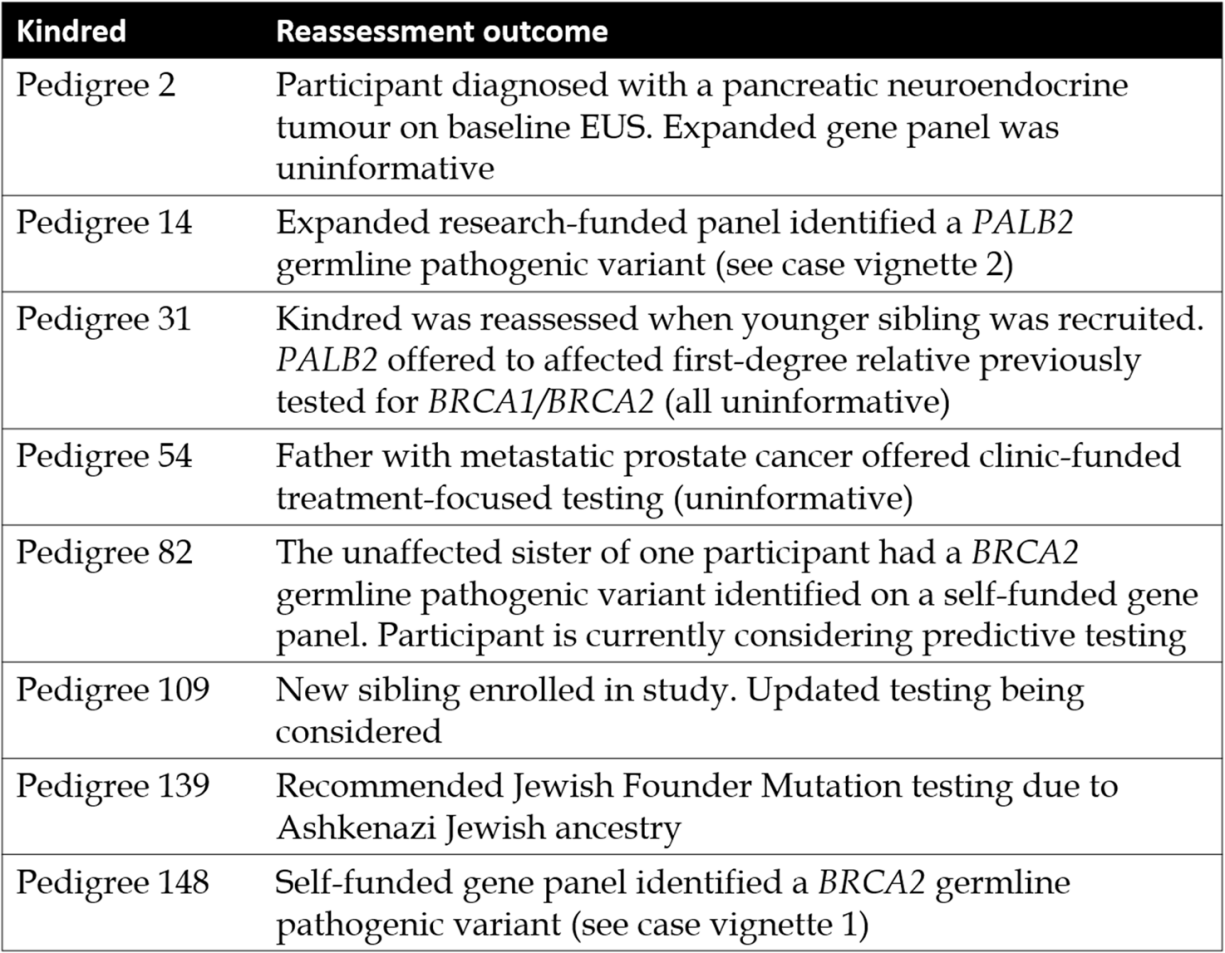
Kindreds reassessed after enrolment

**Table 4:**
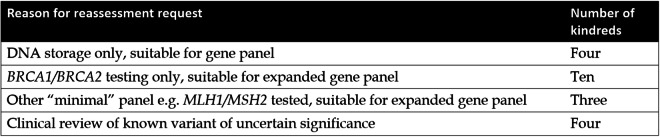
Kindreds identified as suitable for reassessment

### Case vignettes

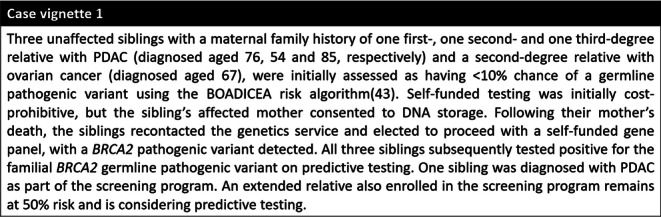

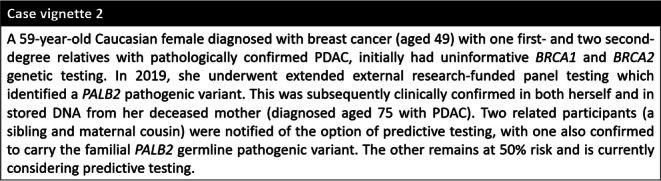





## Discussion

Two hundred and eighty-five high-risk individuals from 189 kindreds participating in the APCSP have undergone mandatory Familial Cancer Service assessment. This is one of the largest PC screening cohorts to have undergone formal genetic counselling, only surpassed by screening studies conducted in countries or regions with a significantly larger population size than Australia [38, 44, 45].

In our study, around 25% of participants (*n* = 71) from 50 kindreds had a known germline pathogenic variant or clinical diagnosis at the time of study enrolment. In addition, 92 participants from 48 kindreds underwent genetic testing after study enrolment. Eight participants from five kindreds were found to have a germline pathogenic variant (*n* = 5 *BRCA2*, *n* = 2 *PALB2*, *n* = 1 *CDKN2A*). Three additional participants are at 50% risk of a newly-identified familial germline pathogenic variant but have not yet undergone predictive testing (n = 2 *BRCA2*; n = 1 *PALB2*). This is a significant detection rate of new germline pathogenic variants in 10.4% of kindreds after study enrolment.

One hundred and nineteen participants from 88 kindreds were not offered genetic testing. For most FPC kindreds this was due to the lack of a suitable person to test and/or because no stored DNA from an affected relative was available. This highlights the importance of facilitating genetic testing at the time of PDAC diagnosis. As previously detailed, studies have shown a significant proportion of PDAC patients have a germline pathogenic variant detected, irrespective of family history or age of diagnosis. When age, family history and Ashkenazi Jewish ancestry are considered, this proportion increases. These findings support the recent ASCO guidelines that those with PDAC be referred for genetic counselling, especially if diagnosed < 60 years of age, have Ashkenazi Jewish ancestry, have a family history of PDAC, a personal or family history of other cancers (particularly breast, ovarian, stomach, bowel and melanoma) and a personal or family history of pancreatitis.

A heritable syndrome cannot be discounted in those without these features. Furthermore, as the cost of genetic testing continues to drop significantly, self-funded genetic testing is increasingly within reach for many patients who may wish to pursue this option given the therapeutic and familial implications. This is demonstrated by the increase in self-funded testing uptake in our cohort within the last five years. As PDAC can unfortunately be associated with rapid deterioration for some, DNA storage should be considered in all. As highlighted in case vignette 1, family members who wish to pursue genetic testing in the future will benefit from an affected relatives’ stored DNA. Increasingly, somatic testing is being arranged by physicians to guide patient therapy. Unfortunately, somatic testing is not adequate for reliable detection of germline pathogenic variants, therefore paired germline testing should be considered [17].

Although genetic testing in the affected individual would be ideal, this is not always feasible. Cremin et al. 2020 [30] noted that when prospective genetic testing for unselected patients with PDAC was offered in British Columbia, Canada, 12.8% (*n* = 39) PDAC patients died before genetic testing could be offered or performed, and overall, only 59.2% (*n* = 177/299) of all referred index patients completed testing despite the presence of efficient testing protocols and the availability of telehealth services. PDAC-related morbidity, mortality and logistic/travel challenges have all been cited in the literature as barriers [46]. Consideration of DNA storage for affected individuals or genetic testing on tumour tissues may offer other avenues of identifying germline pathogenic variants in patients with FPC histories.

A proportion of FPC kindreds in our cohort were not offered genetic testing after Familial Cancer Service review as they did not have a family history characteristic of a heritable cancer syndrome. Of note, at the time of our analysis, there were no national guidelines to offer testing to PDAC affected individuals apart from those with features of an inherited cancer syndrome. In some families current testing criteria may miss relevant germline pathogenic variants [33]. To the authors’ knowledge, only eight kindreds have undergone a subsequent genetics review since the time of their initial Familial Cancer Service assessment. As all participant information was reviewed during this analysis, a further 21/134 kindreds (16%) were identified where additional clinic-funded genetic testing should be considered. Our previous published data indicates that 89% of participants are interested in genetics review as new information becomes available and 80% of untested participants reported wanting to undergo genetic testing [43]. Since testing guidelines are changing rapidly, we highlight the importance of periodic case review of FPC kindreds. At minimum, this would entail updating the family history to record any new cancer diagnoses, reviewing participant genetic testing status and suitability for clinical and/or expanded testing, and an updated discussion about inherited and environmental risks.

As demonstrated in case vignette 1, at initial presentation, the family were assessed to have less than 10% chance of finding a germline pathogenic variant and initially declined self-funded testing. By today’s criteria, the family would likely be offered clinic-funded testing based on their family history. Critically almost half (46.6%) of kindreds in our high-risk screening cohort have not undergone genetic testing. With evidence of unexpected findings accumulating, review of these individuals and consideration of testing where appropriate is warranted.

Notably, of the 48 uninformative kindreds tested so far, only one variant of uncertain significance was identified. All germline pathogenic variants identified were relevant to PDAC. This reflects the strength of the selectivity of the Familial Cancer Services when offering genetic testing in the past. Broadening the criteria to reduce the chance of missing a relevant germline pathogenic variant needs to be balanced against identifying a variant of uncertain significance or a germline pathogenic variant in a less well-established PDAC gene and the significant uncertainty this can generate for individuals and their families [30, 45].

### Limitations

Our study had predominantly Caucasian participants and therefore our findings cannot readily be translated to other ethnic groups. Enrolment in the APCSP first commenced in 2011 with some participants completing genetic counselling many years prior to program enrolment. Therefore, genetic investigations in our cohort occurred over a period of over 20 years as new participants were recruited into the study. The variability in testing performed likely represents the diversity in family histories, in addition to significant changes in testing costs, guidelines and the considerable changes in practice that have occurred over time. Of note, there has been a dramatic increase in the availability of genetic testing options in Australia since 2011, particularly the availability of self-funded testing and broader gene panel testing. However, this is also a relative strength of our study as it reflects real-world experience and the ongoing evolution of standard of care in genetics.

## Conclusion

Germline pathogenic variants associated with PDAC were seen in 29.1% of our high-risk cohort (55/189 kindreds or 82/285 participants). Of these, 5 of 48 (10.4%) kindreds offered mutation analysis were newly-identified to have a germline pathogenic variant after study enrolment. Therefore, in a subset of participants enriched for a family history of PDAC, the rate of new germline pathogenic variants detected was high with *BRCA2* being the most represented. Updated and new gene panel testing should be considered in selected high-risk individuals undergoing PC screening. There are benefits to screening programs periodically analysing their data and rereferral to a Familial Cancer Service should be considered for FPC families who last consulted a genetics service over 5 years ago. A significant proportion of our cohort (29/139 (21%) kindreds) already underwent or would benefit from reassessment by a genetics service. A Familial Cancer Service referral for all newly diagnosed individuals with PDAC should be considered with DNA storage discussed early in their clinical course. The option of treatment-focused and/or self-funded genetic testing should also be discussed as this may help improve clinical outcomes for individuals affected by PDAC and their families.

## Data Availability

The datasets generated and/or analysed during the current study are available from the corresponding author on reasonable request.

## Abbreviations

APCSP: Australian Pancreatic Screening Program
ASCO: American Society of Clinical Oncology
BCC: Basal cell carcinoma
BOADICEA: Breast and Ovarian Analysis of Disease Incidence and Carrier Estimation Algorithm
CAPS: Cancer of the Pancreas Surveillance
CRC: Colorectal cancer
DCIS: Ductal carcinoma in situ
EUS: Endoscopic ultrasound
FDR: First-degree relative
FPC: Familial pancreatic cancer
GPV: Germline pathogenic variant
JFM: Jewish Founder Mutation
MMR IHC: Immunohistochemistry of the mismatch repair proteins
MRI: Magnetic resonance imaging
NATA: National Association of Testing Authorities
PARP: Poly ADP-ribose polymerase
PC: Pancreatic cancer
PDAC: Pancreatic ductal adenocarcinoma
pNET: pancreatic neuroendocrine tumour
RCC: Renal cell carcinoma
SCC: Squamous cell carcinoma
SDR: Second-degree relative
TDR: Third-degree relative
VUS: Variant of uncertain significance
WES: Whole exome sequencing
