# Correction to: Significant detection of new germline pathogenic variants in Australian Pancreatic Cancer Screening Program participants

**DOI:** 10.1186/s13053-021-00195-w

**Published:** 2021-09-08

**Authors:** Krithika Murali, Tanya M. Dwarte, Mehrdad Nikfarjam, Katherine M. Tucker, Rhys B. Vaughan, Marios Efthymiou, Allison Collins, Allan D. Spigelman, Lucinda Salmon, Amber L. Johns, David B. Williams, Martin B. Delatycki, Thomas John, Alina Stoita

**Affiliations:** 1 Department of Clinical Genetics, Austin Health, Heidelberg, VIC 3084 Australia; 2 Australian Pancreatic Cancer Genome Initiative, Garvan Institute of Medical Research, Darlinghurst, NSW 2010 Australia; 3 Hereditary Cancer Centre, Prince of Wales Hospital, Randwick, NSW 2031 Australia; 4 Department of Gastroenterology, St Vincent’s Hospital, Darlinghurst, NSW 2010 Australia; 5 Division of Surgery, Austin Health, Heidelberg, VIC 3084 Australia; 6 University of New South Wales, St Vincent’s Clinical School and Prince of Wales Clinical School, Randwick, NSW 2031 Australia; 7 Department of Gastroenterology, Austin Health, Heidelberg, VIC 3084 Australia; 8 Clinical Trials Unit, Olivia Newton John Cancer and Wellness Centre, Austin Health, Heidelberg, VIC 3084 Australia; 9 Cancer Genetics Unit, The Kinghorn Cancer Centre, St Vincent’s Hospital, Darlinghurst, NSW 2010 Australia; 10 Peter MacCallum Cancer Centre, Parkville, VIC 3000 Australia

**Correction to: Hered Cancer Clin Pract 19, 33 (2021)**

**https://doi.org/10.1186/s13053-021-00190-1**

Following publication of the original article [1], an error was identified in affiliation 6. The correct affiliation 6 is:

University of New South Wales, St Vincent's Clinical School and Prince of Wales Clinical School, Randwick, NSW 2031, Australia

The original article [1] has been corrected.
